# Supplementary material for: plasticity of TGF-β signaling
Source: BMC Syst Biol. 2011 Nov 3;5:184. doi: 10.1186/1752-0509-5-184 (PMC3227652; doi:10.1186/1752-0509-5-184)
Supplement: Additional file 2 — Supplementary_Tables. Tables of the model parameters and the equations used in the model. [file 1752-0509-5-184-S2.PDF]

Supplementary tables
The plasticity of TGF-β signaling

Geraldine Cellière, Georgios Fengos, Marianne Hervé and Dagmar Iber

Table S1: **Model parameters.** Parameter names, units, ranges and literature values with their references.

Table S2: **Reaction equations used in the model.** R-Smad is denoted as Smad, $TGF\beta R$ represents the receptor, an underscore between two species indicates the complex of both species, _P stands for phosphorylated proteins and _N symbolize the nuclear location. When no nuclear location is specified, the name depicts the cytoplasmic species. $c = V_c/V_{ref}$ and $n = V_n/V_{ref}$ account for the volume difference between nucleus and cytoplasm as we work with concentrations. Here V_c and V_n refer to the cytoplasmic and nuclear volumes while V_{ref} is a reference volume.

Table S3 : **Parameters used in Additional file 1, Fig. S8, S9, S10, and S11.** A representative transient and a representative sustained response were selected and the corresponding parameter sets were used to plot the temporal evolution of each species in both cases.

Parameters	Units	Minimum	Maximum	Literature	References
c	-	2.3	2.3	2.3	[1]
n	-	1	1	1	[1]
h		1	4	-	-
k1	s^{-1}	10^{-5}	10^{-2}	2.3×10^{-5} and 5×10^{-3}	$[2, 3]$
k2	$pM^{-1}.s^{-1}$	10^{-7}	10^{-3}	1.5×10^{-4} and 1.54×10^{-4}	$[2, 3]$
k3	s^{-1}	10^{-3}	1	-	-
k4	s^{-1}	10^{-3}	1	-	-
k5	$pM.s^{-1}$	10^{-4}	1	-	-
k6	s^{-1}	10^{-6}	1	-	-
k7	$pM.s^{-1}$	10^{-7}	10^{-5}	4×10^{-7} and 3.5×10^{-6}	$[1, 3]$
k8	s^{-1}	10^{-5}	10^{-1}	$2.6 \times 10^{-3}$and 8.3×10^{-5} and 2.7×10^{-3}	$[1, 3, 4]$
k9	s^{-1}	10^{-5}	10^{-1}	$5.6 \times 10^{-3}$and 9.4×10^{-2} and 5.8×10^{-3}	$[1, 3, 4]$
k10	$pM^{-1}.s^{-1}$	10^{-8}	10^{-4}	$1.6 \times 10^{-5}$and 1.4×10^{-6} and 3.9×10^{-6}	$[1, 3, 4]$
k11	s^{-1}	10^{-4}	1	$1.8 \times 10^{-6}$and 7.5×10^{-4} and 1.5×10^{-2}	$[1, 3, 4]$
k12	-	10^{-2}	10	5.7	[1]
k13	s^{-1}	10^{-3}	10^{-1}	4.2×10^{-4}	[3]
k14	$pM.s^{-1}$	10^{-2}	10^{3}	-	-
k15	pM	1	10^{5}	-	-
k16	s^{-1}	10^{-5}	10^{-1}	-	-
k17	s^{-1}	10^{-5}	10^{-1}	-	-
k18	s^{-1}	10^{-5}	10^{-1}	-	-
k19	s^{-1}	10^{-5}	10^{-1}	-	-
$TGF\beta R$	pM	10^{3}	10^{3}	10^{3}, $4 \times 10^{3}$and 10^{4}	$[1, 2, 4]$
Smad	pM	6×10^{4}	6×10^{4}	1.78×10^{5}, 3.6×10^{5}, $10^{5}$and 1.5×10^{5}	$[1, 2, 4, 5]$
Cosmad	pM	10^{5}	10^{5}	10^{5}, 8.4×10^{5}, $10^{5}$and 1.5×10^{5}	$[1, 2, 4, 5]$
TGF-β	pM	200	200	80	[5]

Table 1: **Model parameters.**

Differential equations	Reactions definitions	
$\frac{d[TGF\beta R]}{dt} = r1 - r2$	$r1 = k1 \times [TGF\beta\_TGF\beta R]$	$r18 = k9 \times [Smad\_P\_N]$
$\frac{d[TGF\beta\_TGF\beta R]}{dt} = -r1 + r2 - r3 + r4 + r6$	$r2 = k2 \times [TGF\beta R] \times [TGF\beta]$	$r19 = k12 \times k8 \times [Smad\_P\_CoSmad]$
$\frac{d[TGF\beta\_TGF\beta R\_P]}{dt} = r3 - r4 - r5$	$r3 = k3 \times [TGF\beta\_TGF\beta R]$	$r20 = k13 \times [Smad\_P\_N]$
$\frac{d[I\_Smad\_TGF\beta\_TGF\beta R\_P]}{dt} = r5 - r6$	$r4 = k4 \times [TGF\beta\_TGF\beta R\_P]$	$r21 = k10 \times [Smad\_P\_N] \times [Smad\_P\_N]$
$\frac{d[Smad]}{dt} = -r7 - r8/c + r9/c$	$r5 = k5 \times [TGF\beta\_TGF\beta R\_P] \times [I\_Smad]$	$r22 = k11 \times [Smad\_P\_Smad\_P\_N]$
$\frac{d[Smad\_P]}{dt} = r7 - r10 + r11 - r12 + r13 - r17/c + r18/c$	$r6 = k6 \times [I\_Smad\_TGF\beta\_TGF\beta R\_P]$	$r23 = k10 \times [Smad\_P\_N] \times [CoSmad\_N]$
$\frac{d[CoSmad]}{dt} = -r12 + r13 - r14/c + r15/c$	$r7 = k7 \times [Smad] \times [TGF\beta\_TGF\beta R\_P]$	$r24 = k11 \times [Smad\_P\_CoSmad\_N]$
$\frac{d[Smad\_P\_Smad\_P]}{dt} = r10 - r11 - r16/c$	$r8 = k8 \times [Smad]$	$r25 = k14 \times \frac{[Smad\_P\_CoSmad\_N]^h}{[Smad\_P\_CoSmad\_N]^h + k15^h}$
$\frac{d[Smad\_P\_CoSmad]}{dt} = r12 - r13 - r19/c$	$r9 = k9 \times [Smad\_N]$	$r26 = k16 \times [I\_Smad\_mRNA1]$
$\frac{d[Smad\_N]}{dt} = r8/n - r9/n + r20$	$r10 = k10 \times [Smad\_P] \times [Smad\_P]$	$r27 = k17 \times [I\_Smad\_mRNA2]$
$\frac{d[Smad\_P\_Smad\_P\_N]}{dt} = r16/n + r21 - r22$	$r11 = k11 \times [Smad\_P\_Smad\_P]$	$r28 = k18 \times [I\_Smad\_mRNA2]$
$\frac{d[Smad\_P\_N]}{dt} = r17/n - r18/n - r20 - r21 + r22 - r23 + r24$	$r12 = k10 \times [Smad\_P] \times [CoSmad]$	$r29 = k19 \times [I\_Smad]$
$\frac{d[Smad\_P\_CoSmad\_N]}{dt} = r19/n + r23 - r24$	$r13 = k11 \times [Smad\_P\_CoSmad]$	
$\frac{d[CoSmad\_N]}{dt} = r14/n - r15/n - r23 + r24$	$r14 = k8 \times [CoSmad]$	
$\frac{d[I\_Smad\_mRNA1]}{dt} = r25 - r26/n$	$r15 = k9 \times [CoSmad\_N]$	
$\frac{d[I\_Smad\_mRNA2]}{dt} = r26/c - r27$	$r16 = k12 \times k8 \times [Smad\_P\_Smad\_P]$	
$\frac{d[I\_Smad]}{dt} = r28 - r29 + r6 - r5$	$r17 = k8 \times [Smad\_P]$	

Table 2: **Reaction equations used in the model.**

Parameters	Transient Response	Sustained Response
h	2.06	1.32
k1	4.46×10^{-3}	4.41×10^{-5}
k2	4.39×10^{-6}	1.47×10^{-6}
k3	3.24×10^{-1}	3.62×10^{-2}
k4	1.92×10^{-3}	1.11×10^{-2}
k5	5.49×10^{-4}	2.40×10^{-1}
k6	1.29×10^{-5}	4.69×10^{-4}
k7	9.35×10^{-6}	6.44×10^{-6}
k8	1.04×10^{-2}	2.05×10^{-3}
k9	7.50×10^{-4}	1.74×10^{-4}
k10	5.12×10^{-8}	2.77×10^{-7}
k11	9.23×10^{-3}	5.61×10^{-3}
k12	5.13×10^{-2}	1.02
k13	1.64×10^{-3}	2.26×10^{-3}
k14	3.80×10^{-2}	2.04×10^{-1}
k15	28.52	1131.8
k16	2.14×10^{-2}	2.74×10^{-4}
k17	8.05×10^{-5}	6.02×10^{-2}
k18	4.34×10^{-2}	1.05×10^{-3}
k19	4.12×10^{-4}	1.21×10^{-5}

Table 3: **Parameters used in Additional file 1, Fig. S8, S9, S10, and S11.**

References

[1] Schmierer B, Tournier AL, Bates PA, Hill CS: **Mathematical modeling identifies Smad nucleocytoplasmic shuttling as a dynamic signal-interpreting system**. *Proc Natl Acad Sci U S A* 2008, **105**(18):6608–13.

[2] Clarke DC, Liu X: **Decoding the quantitative nature of TGF-beta/Smad signaling**. *Trends Cell Biol* 2008, **18**(9):430–42.

[3] Chung SW, Miles FL, Sikes RA, Cooper CR, Farach-Carson MC, Ogunnaike BA: **Quantitative modeling and analysis of the transforming growth factor beta signaling pathway**. *Biophys J* 2009, **96**(5):1733–50.

[4] Clarke DC, Betterton MD, Liu X: **Systems theory of Smad signalling**. *Syst Biol (Stevenage)* 2006, **153**(6):412–24.

[5] Zi Z, Klipp E: **Constraint-based modeling and kinetic analysis of the Smad dependent TGF-beta signaling pathway**. *PLoS One* 2007, **2**(9):e936.
