# Supplementary material for: plasticity of TGF-β signaling
Source: BMC Syst Biol. 2011 Nov 3;5:184. doi: 10.1186/1752-0509-5-184 (PMC3227652; doi:10.1186/1752-0509-5-184)
Supplement: Additional file 3 — Supplementary_Figures. Figures for the response classification of the parameter screen (Fig. S5), the dependance of the damping of oscillations on k2 (Fig. S6), boxplots for the parameter sets that can lead to both transient and sustained responses (Fig. S7), distribution of the maximal output value (Fig. S8), boxplots for parameters with high and low saturation value (Fig. S10), and with high and low maximal value (Fig. S9), boxplots for faithful and unfaithful parameters sets (Fig. S11), the evolution of the concentrations of all species in a representative cases (Fig. S1, S2, S3 and S4). [file 1752-0509-5-184-S3.PDF]

Supplementary figures
The plasticity of TGF-β signaling

Geraldine Cellière, Georgios Fengos, Marianne Hervé and Dagmar Iber

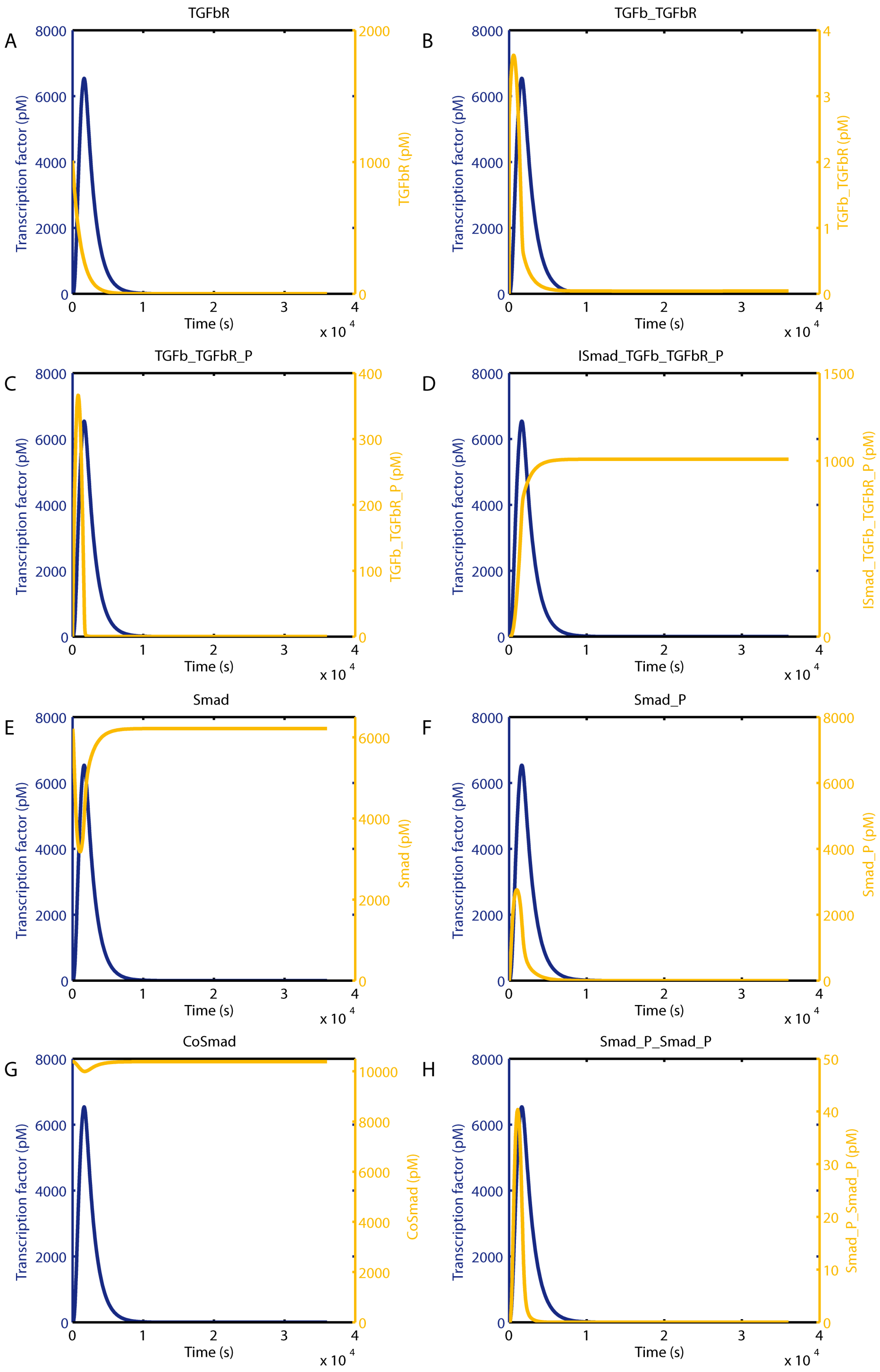

Figure S1: **Evolution of the concentrations of all species in a representative transient response case.** Note that different scales are chosen for each graph. The parameter set used is listed in Additional file 1, Table S3.

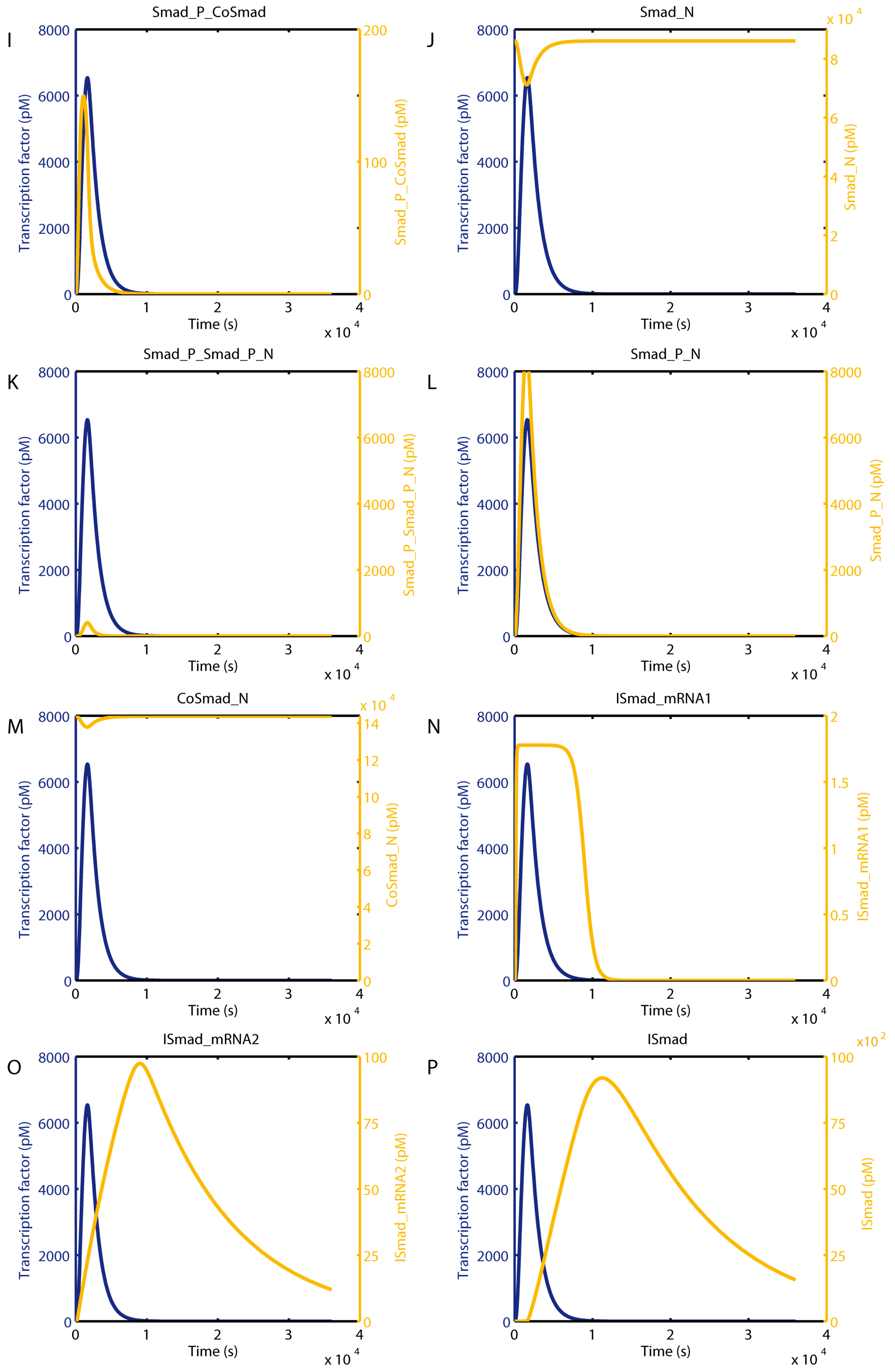

Figure S2: **Evolution of the concentrations of all species in a representative transient response case (continued).** Note that different scales are chosen for each graph. The parameter set used is listed in Additional file 1, Table S3.

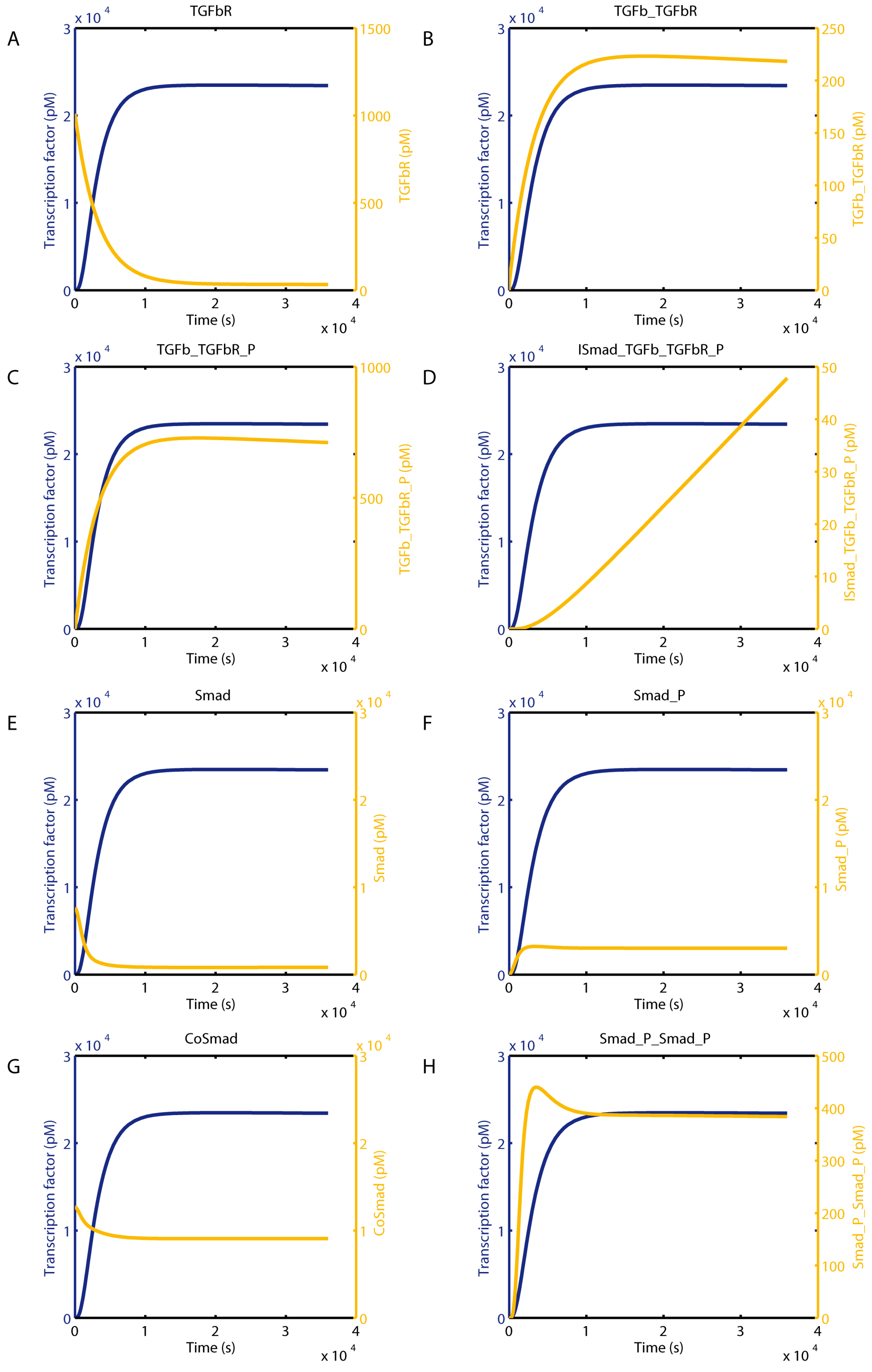

Figure S3: **Evolution of the concentrations of all species in a representative sustained response case.** Note that different scales are chosen for each graph. The parameter set used is listed in Additional file 1, Table S3.

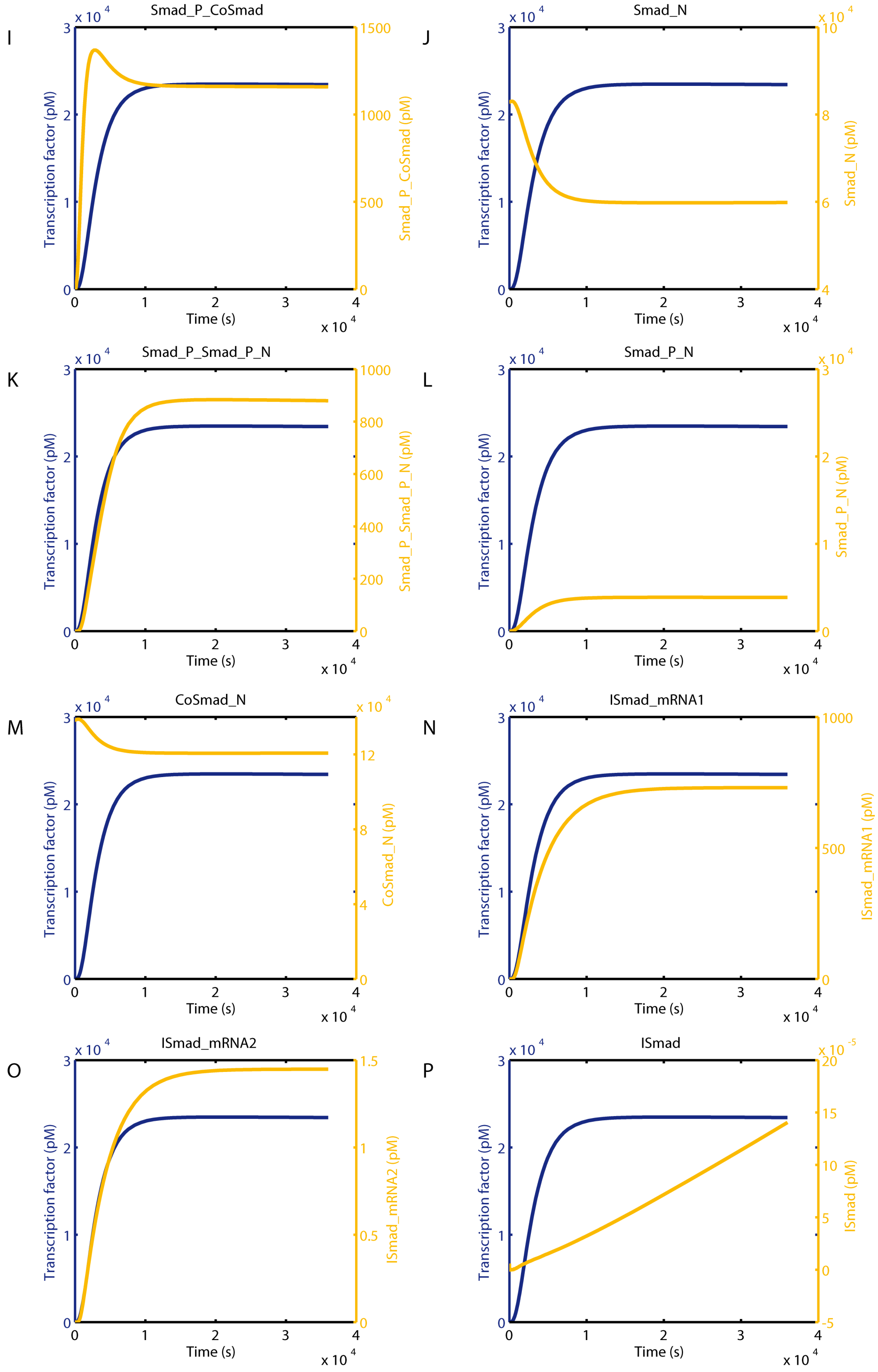

Figure S4: **Evolution of the concentrations of all species in a representative sustained response case (continued).** Note that different scales are chosen for each graph. The parameter set used is listed in Additional file 1, Table S3.

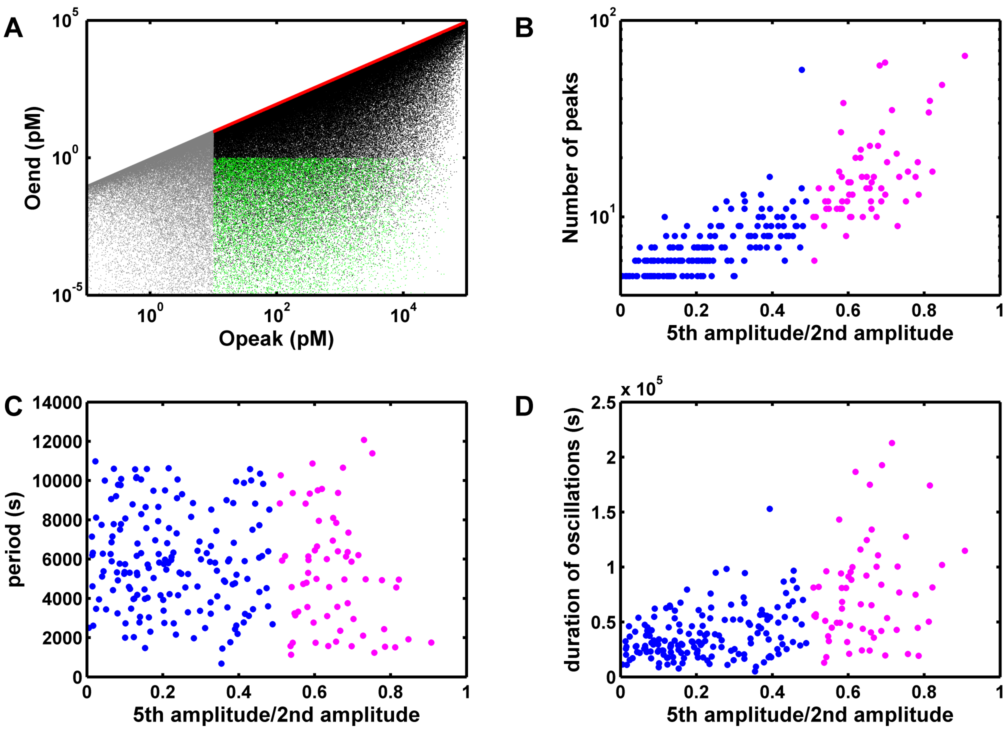

Figure S5: **Response classification of the Parameter screen.** Based on the definitions in Fig. 2 the parameter sets can be classified to give rise to **(A)** sustained (red), transient (green), or no responses (grey) [black parameter sets are undefined according to these criteria], or to **(B)** dampened (blue) or sustained (magenta) oscillations. Dampened oscillations (blue) have lower number of peaks before they completely vanish, compared to more sustained oscillations (purple). **(C)** Sustained (purple) and dampened (blue) oscillations exhibit the same range of periods, and **(D)** the time at which oscillation vanishes (duration) is slightly smaller for dampened oscillations.

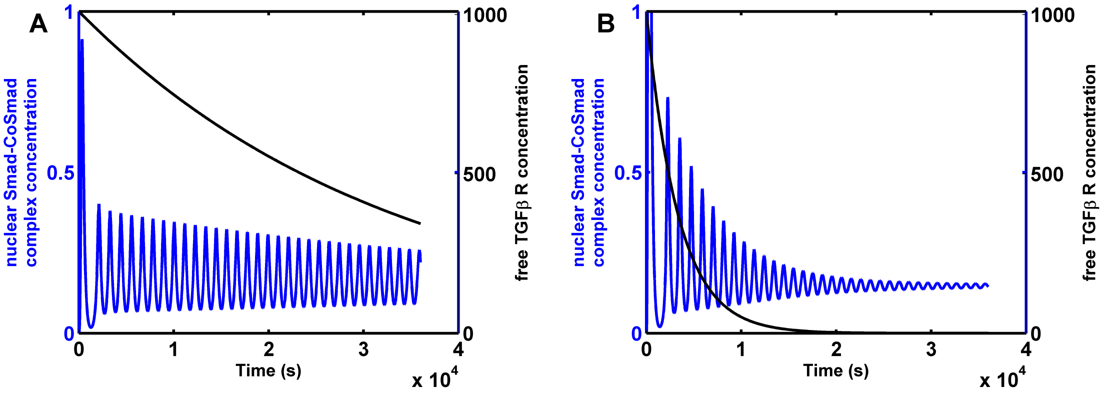

Figure S6: **Damping of oscillations mainly depend on $k2$, the binding rate of TGF-β to its receptor.** **(A)** Transcription factor concentration (blue) and free $TGF\beta R$ (black) evolution over time when $k2$ is small. Oscillations are sustained and the pool of free receptor decreases slowly. **(B)** Transcription factor concentration (blue) and free $TGF\beta R$ (black) evolution over time when $k2$ is 10 times larger than in A. Oscillations are dampened because quickly there is no free receptor available to bind to the ligand to induce a new peak in the transcription factor nuclear concentration.

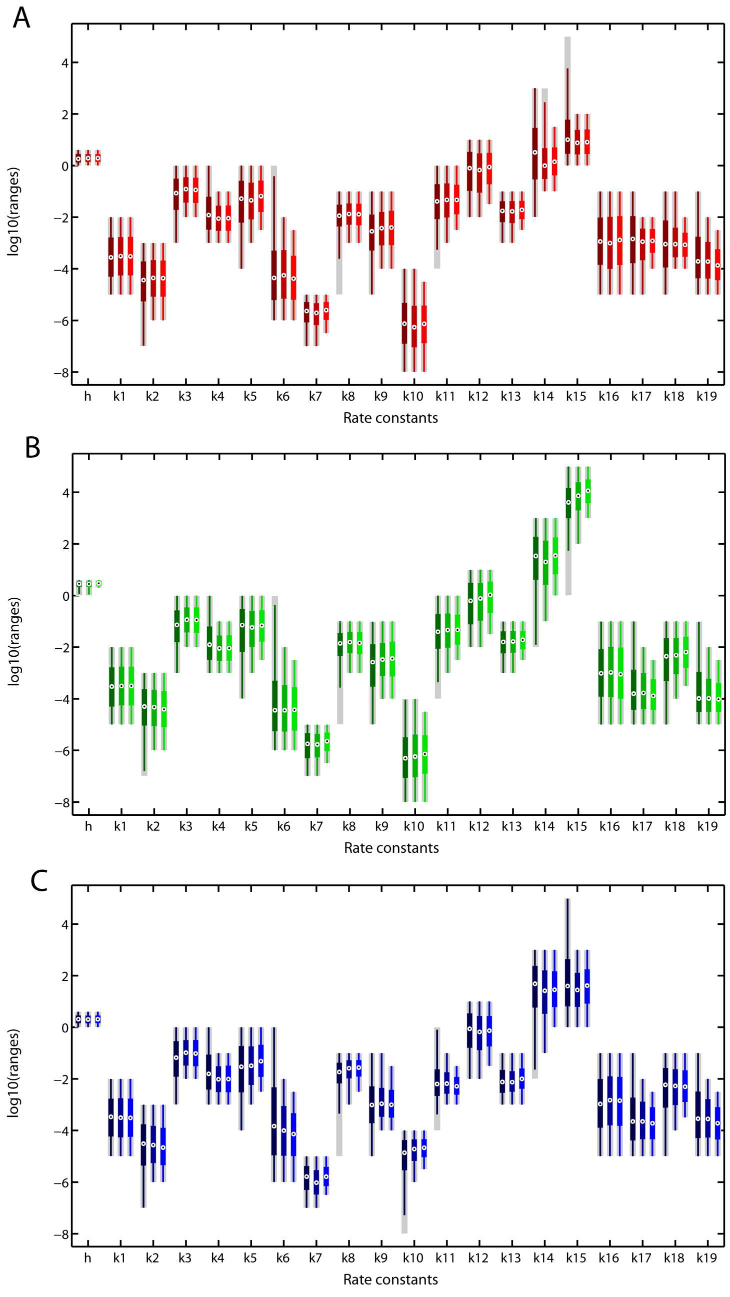

Figure S7: **Parameter sets that can lead to both transient and sustained responses are constrained.** Box plots of parameter sets that can lead to both transient and sustained response when **(A)** $TGF\beta R$, **(B)** R-Smad, or **(C)** Co-Smad concentration is 100 fold higher or lower compared to the value of the initial screening. From the first round (dark colors) to the third round (light colors) the screening ranges are more and more reduced, depending on the boxplot of the previous round, in order to find parameter ranges that maximize the number of parameter sets switching. Ranges of the uniform sampling distributions are indicated by grey boxes.

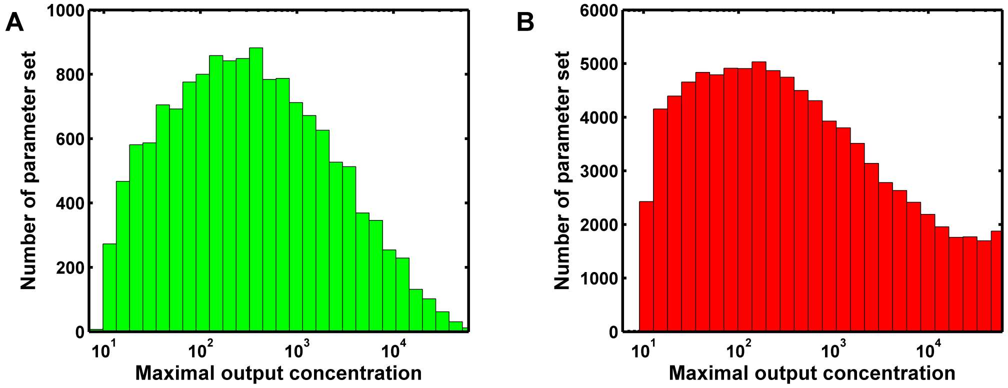

Figure S8: **Distribution of the maximal output value for the transient (A) and sustained (B) ensemble.** Different TGF-β concentration were applied to the system and the maximal value that the output could reach was determined. For more details see Fig. 6.**(A)** The distribution of the maximal output value for the transient ensemble is shown. For most of the sets, this value is comprised between 100 and 1000pM. **(B)** The distribution of the maximal output value for the sustained ensemble is shown. For most of the sets, this value is around 100pM, a bit lower than for the transient set.

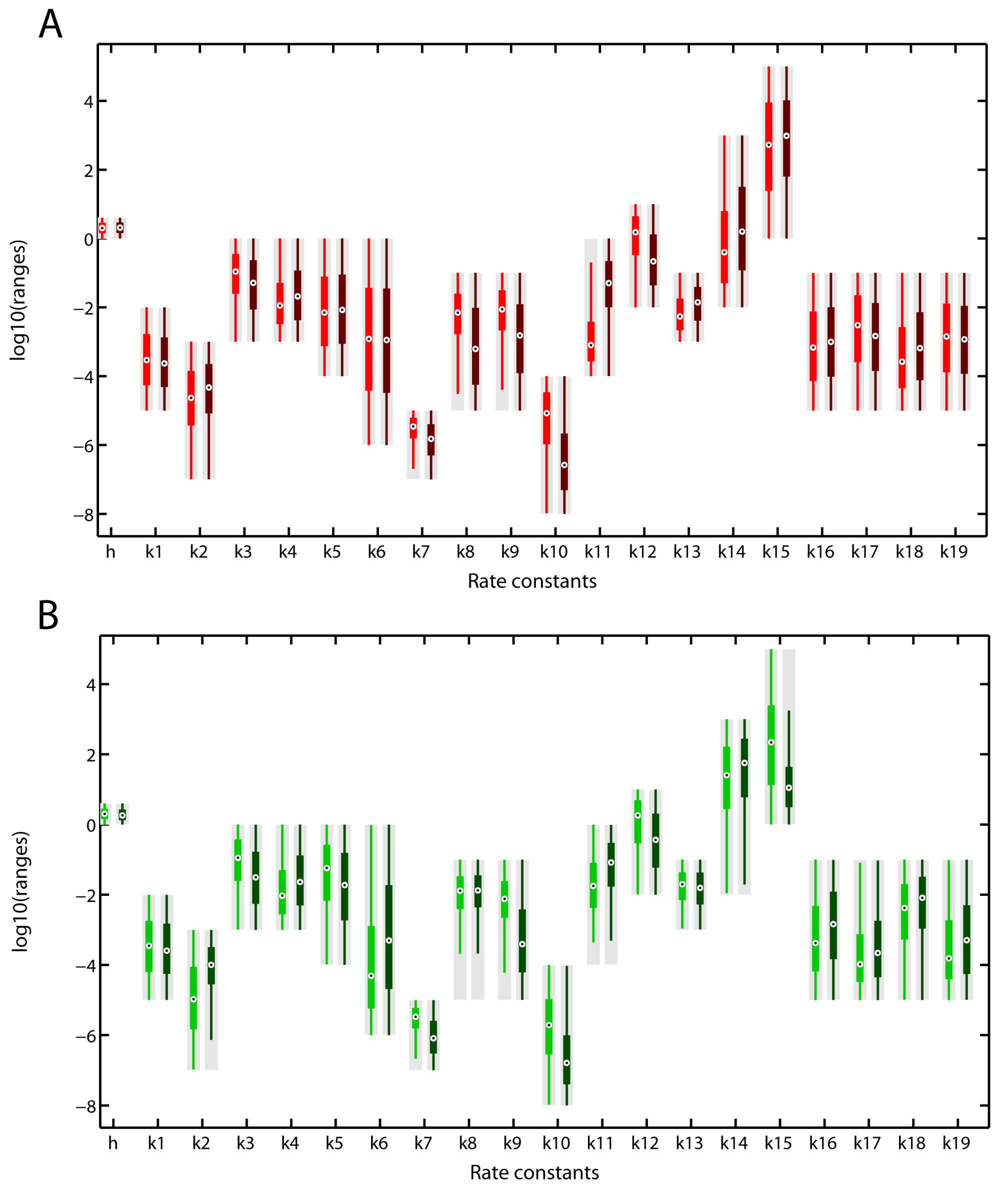

Figure S9: **Box plots of (A) sustained and (B) transient parameter sets with high (light colors) or low (dark colors) maximal reachable output value.** Different TGF-β concentration were applied to the system and the maximal value that the output could reach was determined. For more details see Fig. 6. **(A)** For the parameter set classified as sustained in the initial screening (Additional file 2, Fig. S5), sets with high (light red) or low (dark red) maximal output value are boxplotted. Parameters that differ are mainly $k10$-$k11$ (formation and dissociation of the Smad/Smad and Smad/CoSmad complexes), and $k8$-$k9$ (shuttling rate from cytoplasm into the nucleus and vice-versa). **(B)** For the parameter set classified as transient in the initial screening (Additional file 2, Fig. S5), sets with high (light green) or low (dark green) maximal output value are boxplotted. Parameters that differ are mainly $k2$ (binding of TGF-β to its receptor), $k9$ (shuttling into the cytoplasm), $k10$ (formation of the Smad/Smad and Smad/CoSmad complexes) and $k15$ (I-Smad mRNA synthesis). Ranges of the uniform sampling distributions are indicated by grey boxes.

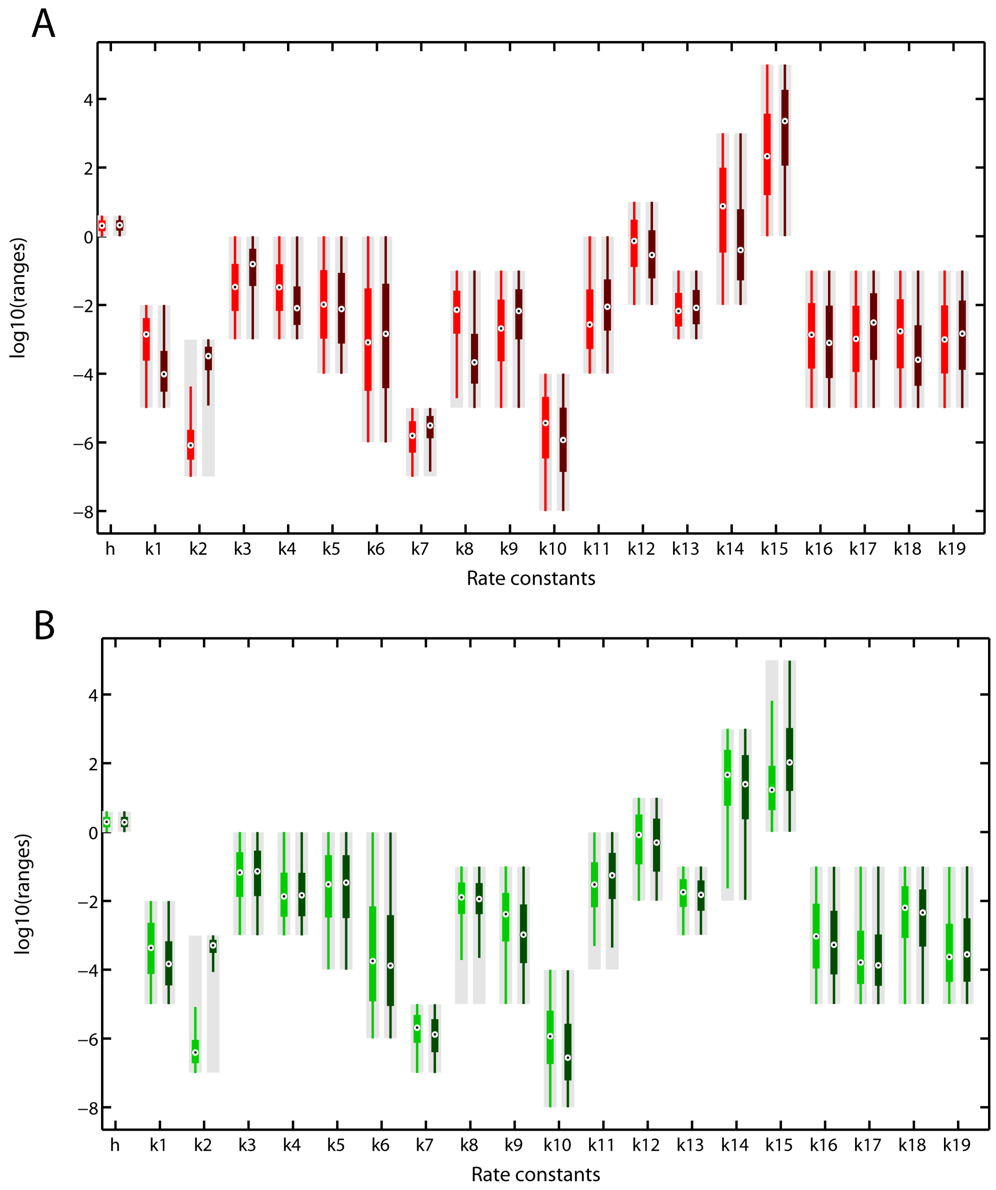

Figure S10: **Box plots of (A) sustained and (B) transient parameter sets with high (light colors) or low (dark colors) TGF-β saturation concentration.** Different TGF-β concentration were applied to the system and the ligand concentration above which the output peak value cannot increase anymore was determined. For more details see Fig. 6. **(A)** For the parameter set classified as sustained in the initial screening (Additional file 2, Fig. S5), sets with high (light red) or low (dark red) TGF-β saturation concentration are boxplotted. Parameters that differ are mainly $k1$-$k2$ (affinity of TGF-β for its receptor), $k3$-$k4$ (phosphorylation affinity of the receptor bound to TGF-β), and $k8$ (shuttling rate into the nucleus). **(B)** For the parameter set classified as transient in the initial screening (Additional file 2, Fig. S5), sets with high (light green) or low (dark green) TGF-β saturation concentration are boxplotted. The only parameter that differs significantly is $k2$ (the binding rate of TGF-β to the receptor). Ranges of the uniform sampling distributions are indicated by grey boxes.

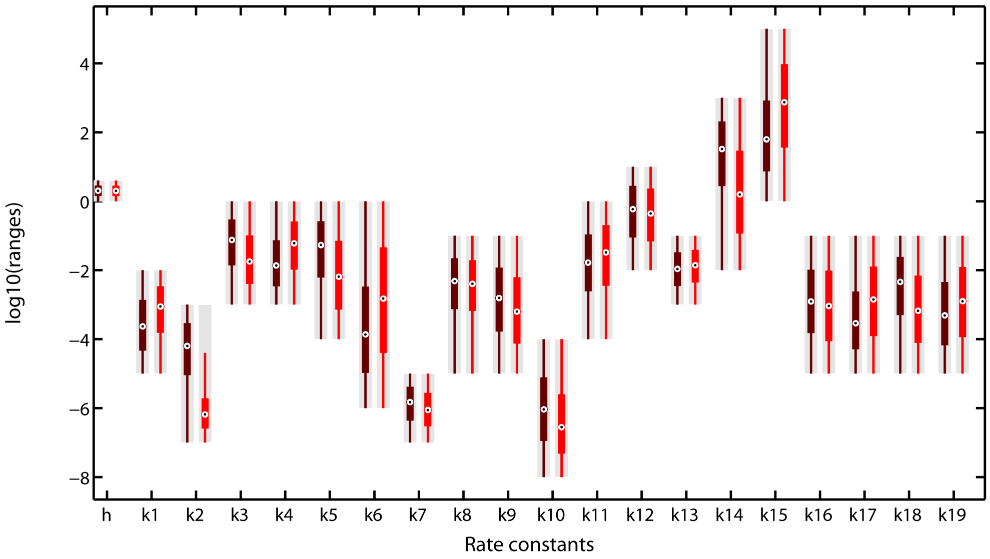

Figure S11: **Box plots of faithful (light red) and unfaithful (dark red) parameter sets.** Procedure to classify the parameter sets into faithful or unfaithful is explained in Fig. 7A. Parameters that differ are mainly $k1$-$k2$ (affinity of TGF-β for its receptor), $k3$-$k4$ (phosphorylation affinity of the receptor bound to TGF-β), $k5$-$k6$ (affinity of the inhibitor for the receptor bound to TGF-β) and $k15$, $k17$, $k18$, $k19$ (all related to the strenght of the feedback). Ranges of the uniform sampling distributions are indicated by grey boxes.
